# Author Correction: Loss of *NARS1* impairs progenitor proliferation in cortical brain organoids and leads to microcephaly

**DOI:** 10.1038/s41467-021-21448-1

**Published:** 2021-02-15

**Authors:** Lu Wang, Zhen Li, David Sievert, Desirée E. C. Smith, Marisa I. Mendes, Dillon Y. Chen, Valentina Stanley, Shereen Ghosh, Yulu Wang, Majdi Kara, Ayca Dilruba Aslanger, Rasim O. Rosti, Henry Houlden, Gajja S. Salomons, Joseph G. Gleeson

**Affiliations:** 1 Department of Neurosciences, Howard Hughes Medical Institute, University of California San Diego, La Jolla, CA 92093 USA; 2 Rady Children’s Institute for Genomic Medicine, Rady Children’s Hospital, San Diego, CA 92123 USA; 3 Metabolic Unit, Department of Clinical Chemistry, Amsterdam UMC, Vrije Universiteit Amsterdam, Amsterdam Neuroscience, Amsterdam Gastroenterology & Metabolism, Amsterdam, Netherlands; 4 Department of Pediatrics, University of California San Diego, La Jolla, CA 92093 USA; 5 Division of Child Neurology, Rady Children’s Hospital, San Diego, CA 92123 USA; 6 Laboratory of Biomanufacturing and Food Engineering, Institute of Food Science and Technology, Chinese Academy of Agricultural Sciences, Beijing, 100193 PR China; 7 University of Tripoli, Tripoli Children’s Hospital, Tripoli, Libya; 8 Department of Medical Genetics, Koç University Hospital, Istanbul, 34010 Turkey; 9 Department of Neuromuscular Disorders, UCL Institute of Neurology, Queen Square, London, WC1N 3BG UK

**Keywords:** Development, Genetics research, Stem-cell research, Neurological disorders

Correction to: *Nature Communications* 10.1038/s41467-020-17454-4, published online 12 August 2020.

The original version of this Article omitted a reference to another publication which included overlapping genetic and MRI data for a research participant. This has been added as reference 59 at the end of the Discussion: ‘While this paper was under review, a separate paper appeared reporting that mutations in *NARS1* associate with neurodevelopmental delay through either biallelic loss or dominant negative effects, impairing NARS1 enzyme activity. This article contained genetic data on family MIC-1433 which overlaps this study^59^.’

Accordingly, reference 59 has now been included in the References section as ‘Manole, A. et al. De novo and bi-allelic pathogenic variants in NARS1 cause neurodevelopmental delay due to toxic gain-of-function and partial loss-of-function effects. *Am. J. Hum. Genet*. **107**(2), 311–324 (2020).’

In addition, the original version of this Article contained an error in Fig. 1A, where there was an error in the family depiction in generation 1 of pedigree MIC-1433; this has been revised to correctly depict the family. Figure 1D also contained MRI scans that had been previously published in Ref 59; this has been revised to include different and unpublished MRI scans from the same individual. The correct version of Fig. 1 is

which replaces the previous incorrect version:

This has been corrected in both the PDF and HTML versions of the Article.

