# Supplementary material for: Loss of NARS1 impairs progenitor proliferation in cortical brain organoids and leads to microcephaly
Source: Nat Commun. 2020 Aug 12;11:4038. doi: 10.1038/s41467-020-17454-4 (PMC7424529; doi:10.1038/s41467-020-17454-4)
Supplement: Supplementary file 1 — Supplementary Info [file 41467_2020_17454_MOESM1_ESM.pdf]

Supplementary Information

Loss of *NARS1* impairs progenitor proliferation in cortical brain organoids and leads to microcephaly

Wang et al.

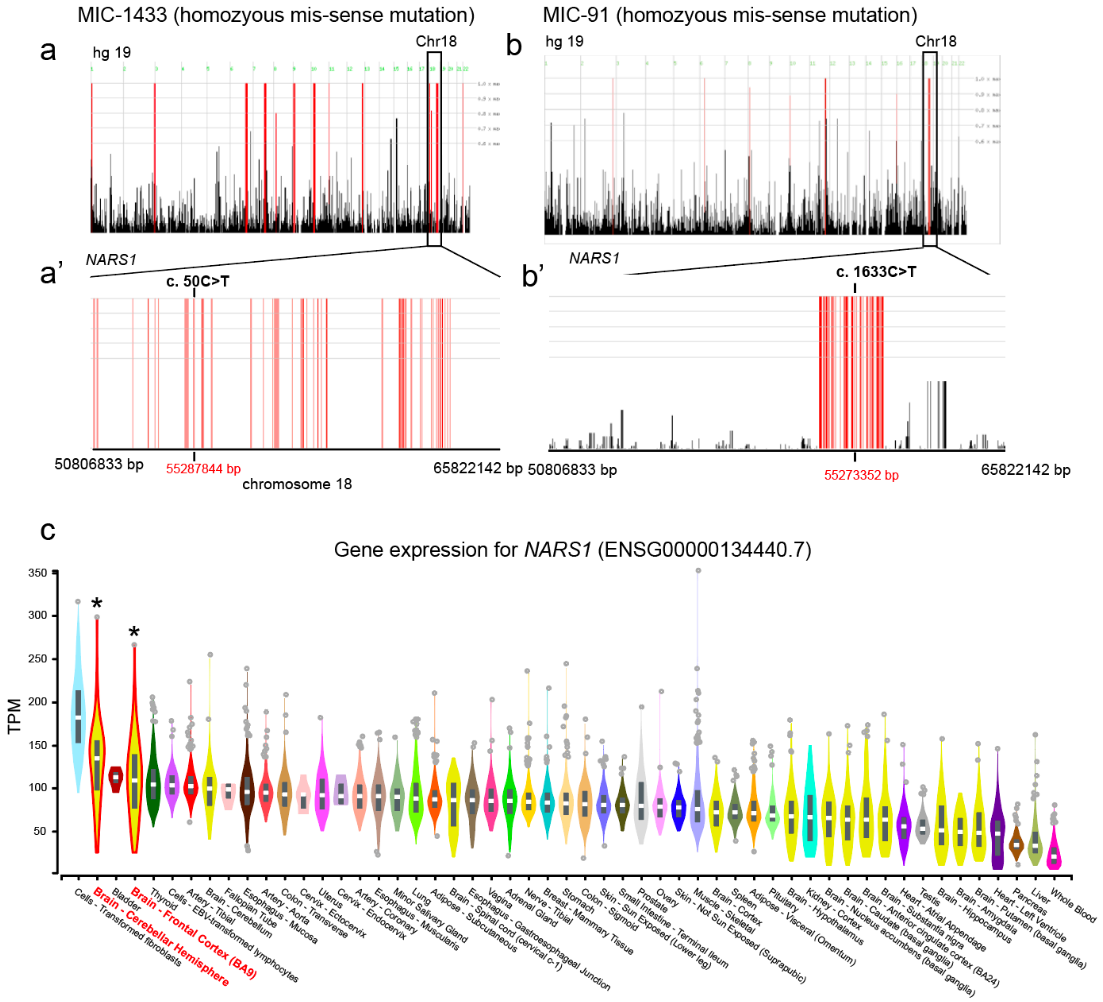

Supplementary Fig. 1: Genetic information for *NARS1* variants. **a-b'.** Homozygosity map showing NARS1 variants falling within blocks of homozygosity Family MIC-1433 and Family MIC-91**.** Family-2116 is not included here because there was no history of parental consanguinity. **c.** GTEX data indicating elevated *NARS1* expression in Brain-Cerebellar Hemisphere and Brain-Frontal Cortex (outlined in red). * indicates the expression in brain related tissues.

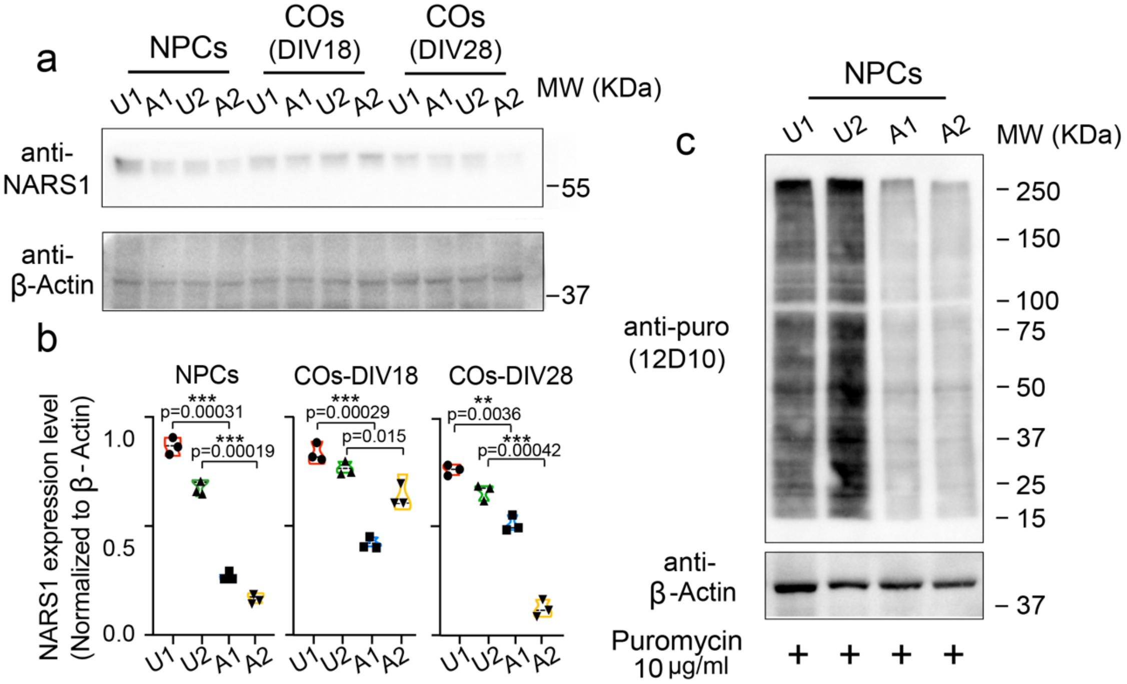

Supplementary Fig. 2: Reduced NARS1 expression in patient cells derived NPCs and COs at different differentiation time points. **a.** Protein expression level of NARS1 during COs differentiation**.** NPCs, COs-DIV18 and COs-DIV28 from two unaffected (U1 and U2) and two affected (A1 and A2) were harvested for WB against anti-NARS1 and actin loading control. **b.** Quantification for WB results of (**a)** showing reduced NARS levels in patient cells. n=3 represents three independent biological replicates, Error bar: ± SD, Student *t*-test with Holm-Šídák multiple comparison correction was used to determine the two-tailed *p*-value, ***p < 0.001, **p < 0.01**.** The individual *p*-values were shown in Supplementary Fig.2**b**. **c.** NPCs were treated with 10μg/ml of puromycin for 1h pulse before harvesting for WB using anti-puromycin antibody (12D10) and actin loading control, demonstrated reduced protein synthesis on cells from affected patients. Source data are available in the Source Data files.

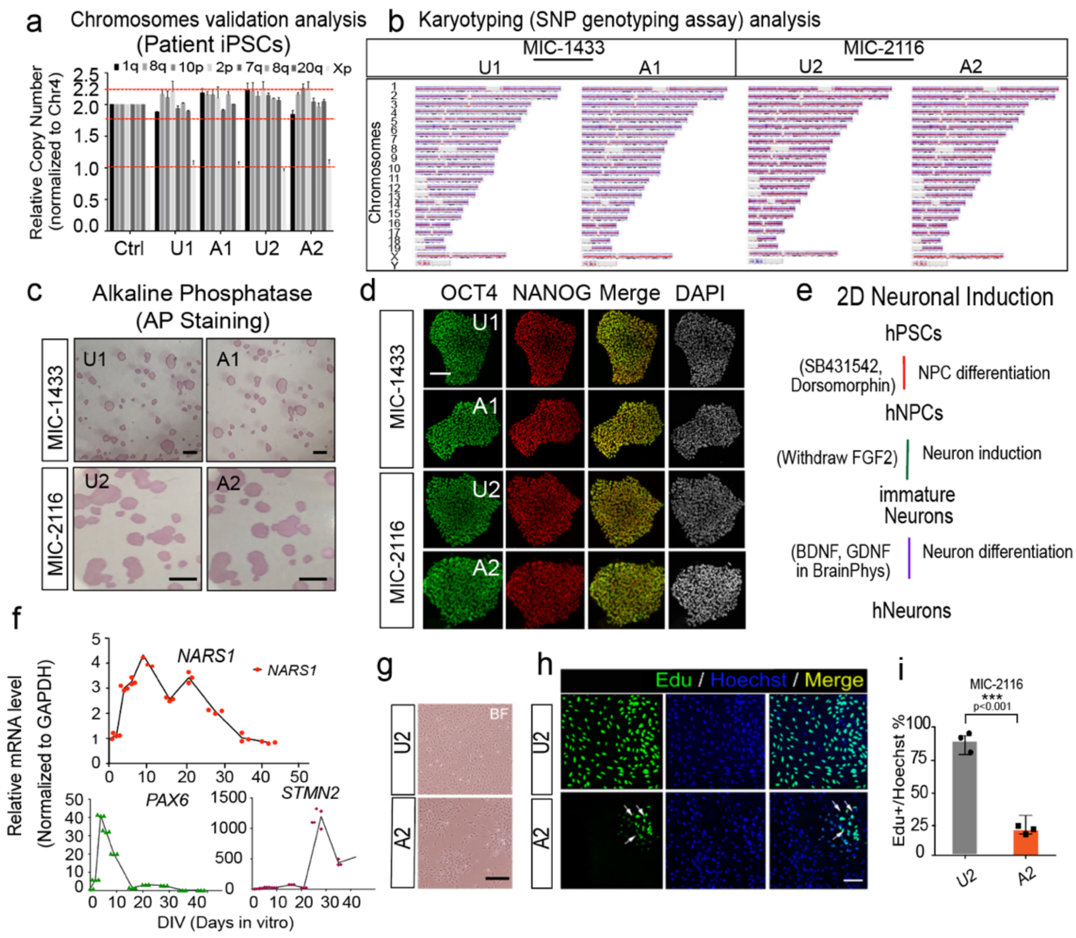

Supplementary Fig. 3: Reduced neuronal proliferation in patient-derived cells in 2D. **a.** Patient-derived iPSCs show chromosome stability. Affected iPSCs were harvest for genomic qPCR to measure chromosomes copy number. Statistic according to Genomic DNA Kit (see in Method). **b.** SNP genotyping assay analysis showing biallelic SNP distribution and absence of karyotypic abnormalities. Grey: position of centromere. **c.** Alkaline Phosphatase (AP) staining for pluripotency of iPSCs. Scale bar = 100 μm for U1/A1 and Scale bar = 200 μm for U2/A2. **d.** Staining of the pluripotency marker of OCT4/NANOG expression of patient iPSCs. Scale bar = 400 μm. **e.** Schematic of 2D neuron generation. **f.** *NARS1* mRNA level increased during early neuron differentiation stages then decreased, compared with *PAX6* and *STMN2* was measured to mark the neuron generation; *GAPDH* was used as control, n=3. **g-h.** Affected NPCs show less differentiation potency and a reduced pluripotency. (**g**) NPCs induced to differentiation, then 1h 10μM Edu pulse with immunostaining and Hoechst; Scale bar = 100 μm, arrows: Edu+ cells in affected (**h**). **i.** Quantification for Edu^{+} cells; n=3, indicates 3 independent biological replicates, Student *t*-test with Holm-Šídák multiple comparison correction was used to determine the two-tailed *p*-value. Error bar: ± SD; Data are presented as mean values ±SD. Source data are available in the Source Data file.

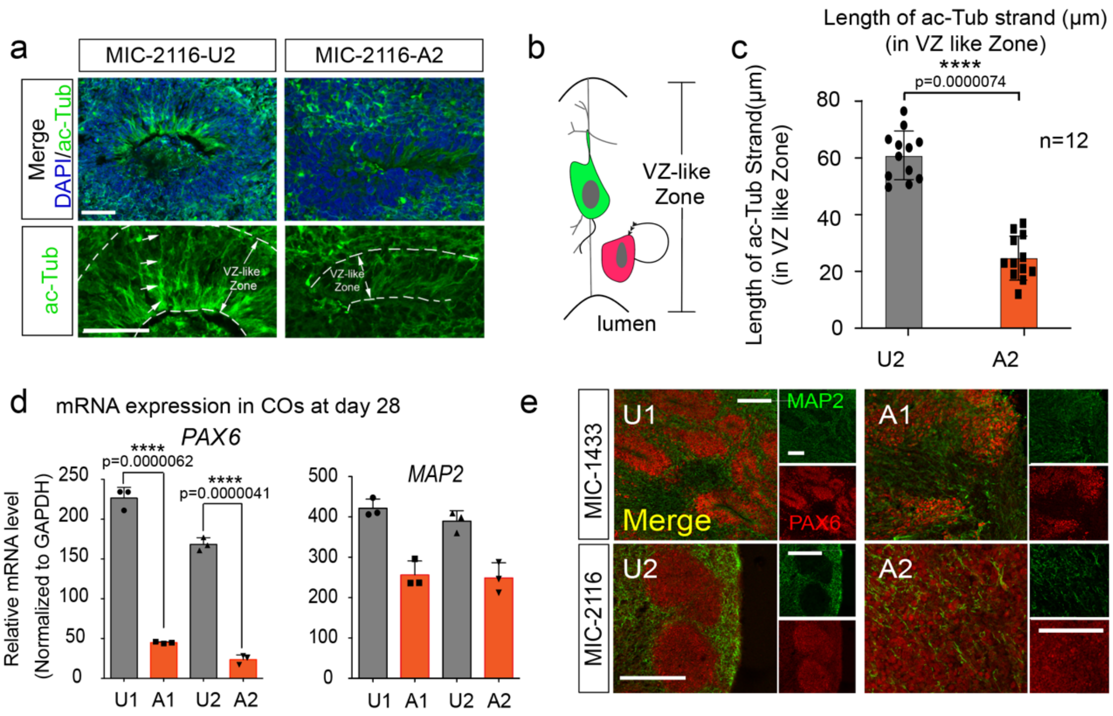

Supplementary Fig. 4: *NARS1* patient COs show reduced size and impaired differentiation **a.** COs were harvested for immunostaining against anti-Acetylated Tubulin (ac-Tubulin), Scale bar = 400 μm. **b.** The schematic shows the cellular composition in VZ-like zone; **c.** The quantification of **c**. n=12 (n represents 3 independent biological replicates generated from iPSCs at different passages (less than 25), and four times technical replicates for each of the independent biological replicate), Student *t*-test with Holm-Šídák multiple comparison correction was used to determine the two-tailed *p*-value, ****p<0.00001, p=0.0000074. Error bar: ± SD. Data are presented as mean values ±SD. **d.** *PAX6* and *MAP2* mRNA level were decrease in affected COs at differentiation day 28. COs were harvested for RNA followed by qPCR. GAPDH was used as negative control, n=3, Error bar: ± SD, Student *t*-test with Holm-Šídák multiple comparison correction was used to determine the two-tailed *p*-value, ****p<0.00001; The individual *p*-values are shown in Supplementary Fig. **4d**. Data are presented as mean values ±SD. **e.** COs were harvested for immunostaining against anti-PAX6 and anti-MAP2, Scale bar = 100 μm. Source data are available in the Source Data file.

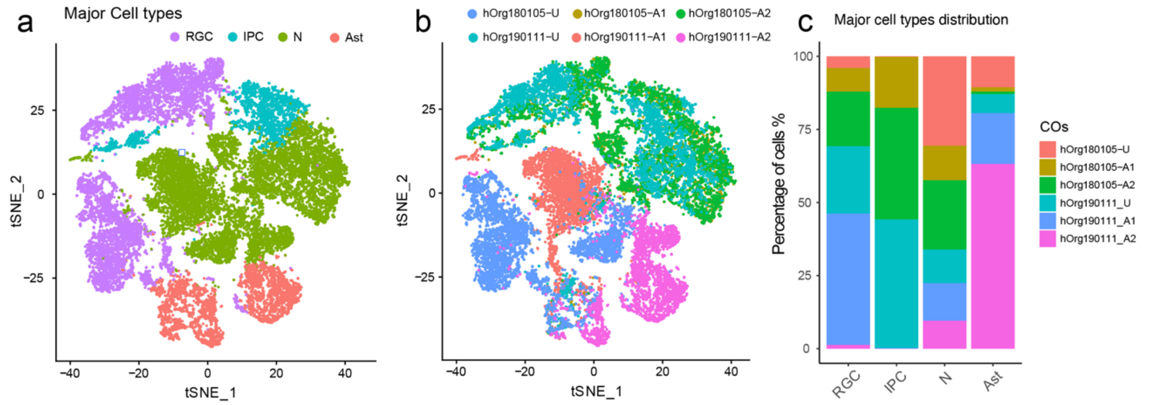

Supplementary Fig. 5: Cell type distribution in different sample replicates for sc-RNA-seq **a.** tSNE plot shown 4 major cell type clusters within the scRNA-seq dataset; **b.** tSNE plot shown the distributions of different samples replicates; **c.** Quantification of major cell type distributions in different sample replicates;

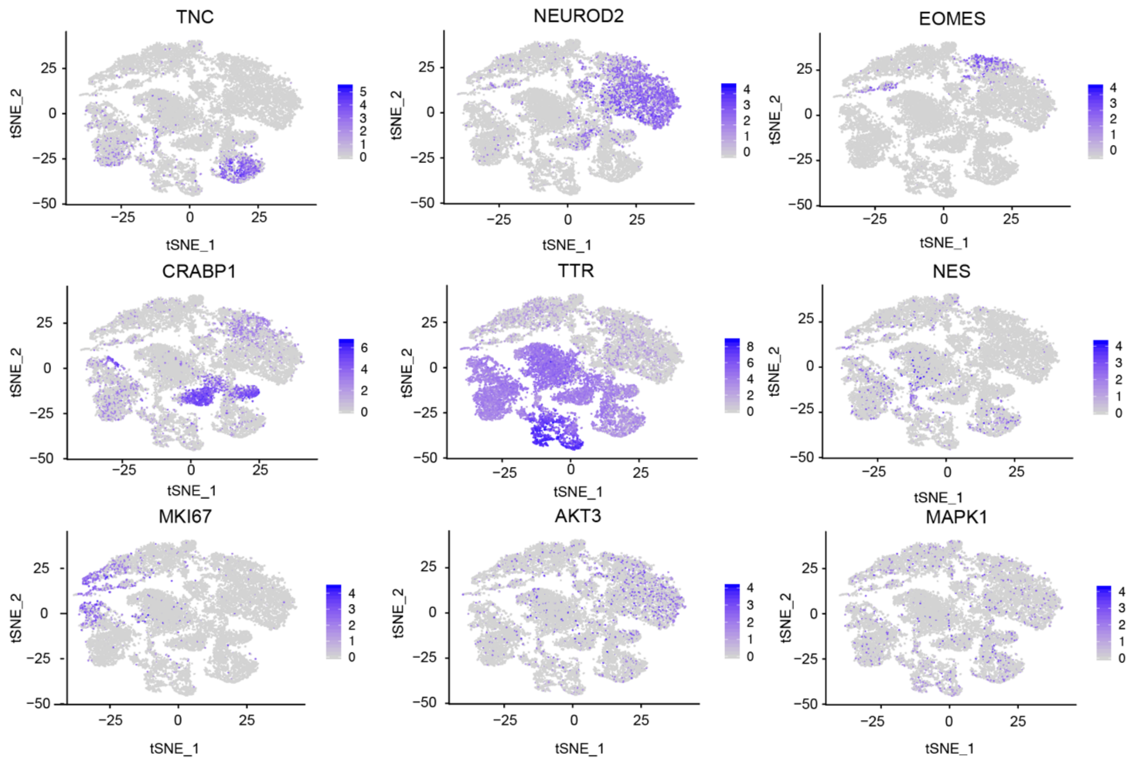

Supplementary Fig. 6: t-SNE plot show expression of different marker genes. t-SNE plot shows the expression of representative marker genes in cell clusters from sc-RNA-seq.

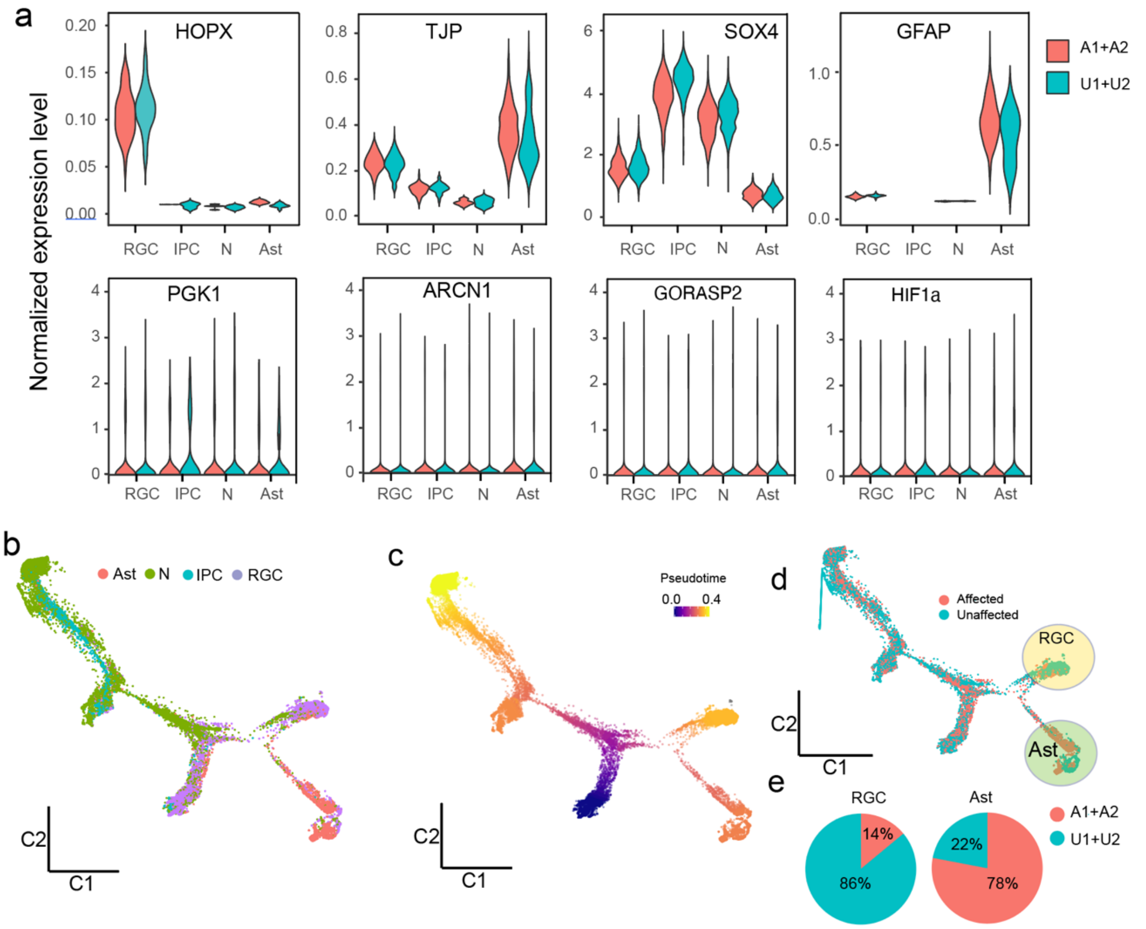

Supplementary Fig. 7: Pseudotime analysis indicated altered cell fates of RGCs and Astrocytes in affected-COs. **a.** Violin-Plot show different gene expression among 4 major different cell clusters; Ast: astrocyte; N: neurons; IPC: intermediate progenitor cells; RGC: radial glia cells. Y axis represents the normalized log UMI counts per cells. **b-c.** Pseudotime analysis indicate cell fate determination in a pseudo-time frame, different cell clusters showed in **b**; and time frame showed in **c**; d. Pseudotime time analysis show cell fate determination between affected and unaffected COs; **e.** Pie plot shows less RGCs and more Astrocytes in the affected COs compared unaffected; RGC: radial glia cell; Ast: astrocyte;

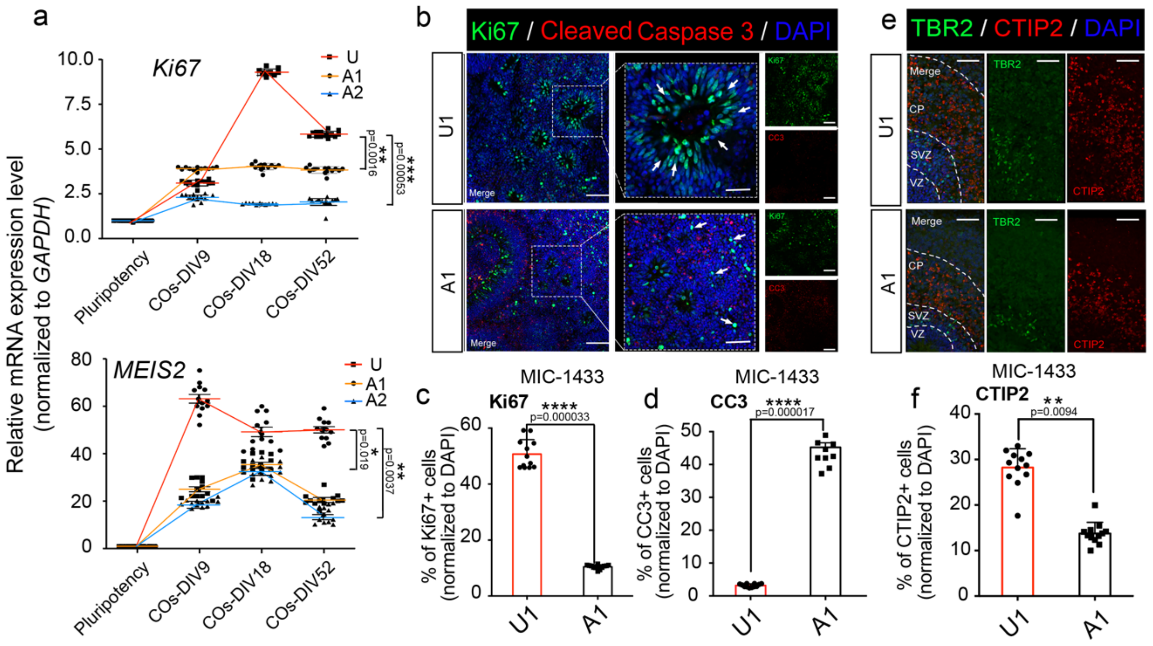

Supplementary Fig. 8: Affected-COs show less proliferation cells and less organized layer formation. **a.** mRNA level of *Ki67* and *MEIS2* during CO development. COs at different DIVs were harvested for RNA and following qPCR to check the expression of *Ki67* and *MEIS2*. n=12 indicates 3 intendent biological replicates and 4 times of technical replicates; Error bar: ± SD, Student *t*-test with Holm-Šídák multiple comparison correction was used for statistical comparison for CO-DIV18, *p<0.1, **p<0.01, ***p<0.001. The exact p-values were shown in Supplementary Fig. **8a**. Data are presented as mean values ±SD. **b.** Ki67 was significantly decreased and CC3 was obviously accumulated in affected CO in Family 1433; COs from both U1 and A1 were harvested for immunostaining against anti-KI67 and anti-Cleaved Caspase 3, DAPI was used to label nucleus. Scale bar = 100 μm **c.** Quantification of $KI67^{+}$ cells in **b**; **d.** Quantification of $CC3^{+}$ cells in **b**; For **c**, and **d**, n=12 indicates 3 intendent biological replicates and 4 times of technical replicates; Error bar: ± SD, Student *t*-test with Holm-Šídák multiple comparison correction was used to determine the two-tailed *p*-value, ****p<0.0001, **p<0.01; The exact *p*-values for **c** is 0.000033, **d** is 0.000017. Data are presented as mean values ±SD. **e.** CTIP2 was significantly decreased in affected CO in Family 1433. Scale bar 100 μm; **f.** Quantification of $CTIP2^{+}$ cells in **e**. n=12 indicates 3 intendent biological replicates and 4 times of technical replicates; Student *t*-test with Holm-Šídák multiple comparison correction was used to determine the two-tailed *p*-value, **p<0.01; The exact *p*-value is 0.0094; Error bar: ± SD. Data are presented as mean values ±SD. Source data are available in the Source Data file.

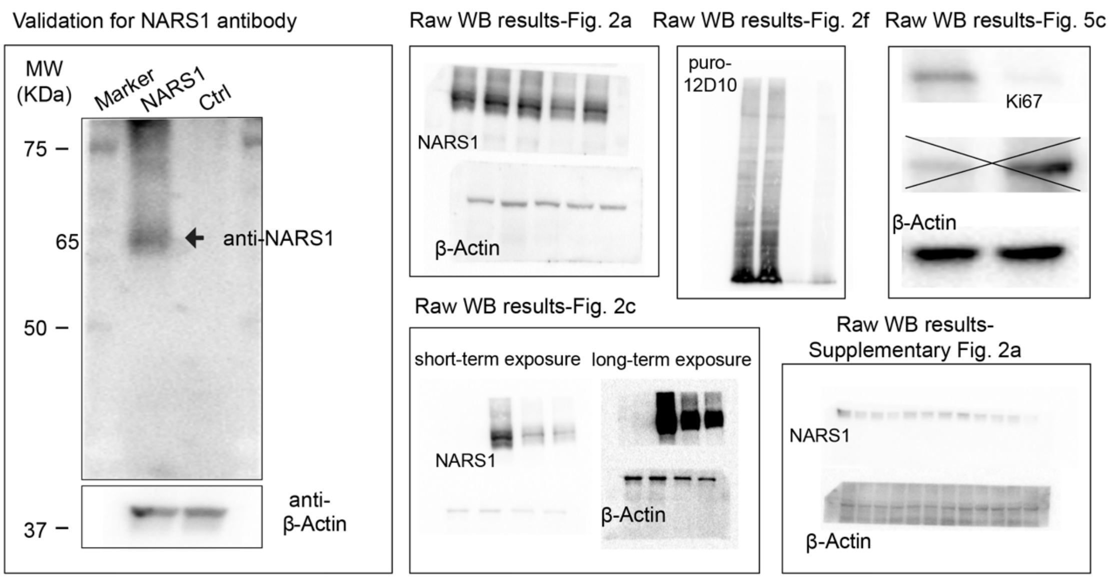

Supplementary Fig. 9: NARS1 antibody validation and raw data for WB shown in manuscript. Raw Western Blot results for Fig 2, 5 and supplementary Fig. 2. “X” indicates the none related band. Source data are available in the Source Data file.

Supplementary Table 1*:* Primers used for different experiments.

Primers sequence for Sanger sequencing in patient cells	
Primers for Sanger sequencing (Fig.1)	
NARS1 (c. 50C>T)-Forward	5’-TGGTTTCTCCTTGGTTCCAT-3’
NARS1 (c. 50C>T)-Reverse	5’-ATTGAAATGGAGTGGGGAAA-3’
NARS1 (c. 1067A>C)-Forward	5’-AGCTCACCAAGCCATCAAAT-3’
NARS1 (c. 1067A>C)-Reverse	5’-AACCAGTGCAGGAGAAAAGC-3’
NARS1 (c. 203dupA)-Forward	5’-CTTTTCCCGGGATTCACTCT-3’
NARS1 (c. 203dupA)-Reverse	5’-CAACCAGTCACTGTGCCAAT-3’
Primer sequence (5’ to 3’) for NARS1 constructs	
NARS1-T17M-Forward	5’-ACACAAGTAGAAGGTGGTACCACACTCTTCAAGCT-3’
NARS1-T17M-Reverse	5’-AGCTTGAAGAGTGTGGTACCACCTTCTACTTGTGT-3’
NARS1-D356A-Forward	5’-GGGCTGGGAGGCACGTCTCCAAGTACA-3’
NARS1-D356A-Reverse	5’-TGTACTTGGAGACGTGCCTCCCAGCCC-3’
Primer sequence (5’ to 3’) for qPCR	
NARS1-Forward	5’-GAACCTGGAAGAAGCAAAGA-3’
NARS1-Reverse	5’-TTATCTTCAGTGTGTCTTGGC-3’
PAX6-Forward	5’-CAGACACAGCCCTCACAAA-3’
PAX6-Reverse	5’-TCATAACTCCGCCCATTCAC-3’
*MAP*2-Forward	5’-CTCTCGCACAGAGTTATCCATC-3’
*MAP*2-Reverse	5’-GACCTACCACCAAGTCCTAAAC-3’
GAPDH-Forward	5’-ACCACAGTCCAT GCCATCAC-3’
GAPDH-Reverse	5’-TCCACCACCCTGTTGCTGTA-3’
NESTIN-Forward	5’-CCATAGAGGGCAAAGTGGTAAG-3’
NESTIN-Reverse	5’-GTGTCTCATGGCTCTGGTTT-3’
KI67-Forward	5’-GCTGAGAACTCCTAAGGGAAAG-3’
KI67-Reverse	5’-GCTGTGAAGCTCTGTAGGATAC-3’
CCND2-Forward	5’-TTCCCTCTGGCCATGAATTAC-3’
CCND2-Reverse	5’-AAACTCAAAGAGACCAGCCC-3’
STMN2-Forward	5’-CTTGAAGCCACCATCTCCTATC-3’
STMN2-Reverse	5’-AAAGAAGAAAGTCTCAGGAGGC-3’
MESI-Forward	5’-ACTTCTGCCACCGATACATTAG-3’
MESI-Reverse	5’-GGGTTATGGTCAGCGAGATT-3’

All the primer sequences are targeted for human sequencing.

Supplementary Note 1

Detailed Sequencing Variant Filtering and Prioritization

Families 2116, 1433

Blood DNA was extracted using Qiagen reagents (Qiagen Inc., USA), then subjected to exome capture with either the Agilent SureSelect Human All Exome 50 Mb kit (Agilent Technologies, Inc., USA) or the Illumina Rapid Capture 37 Mb Enrichment kit. Sequencing with 100-bp paired-end reads was performed using either the Illumina HiSeq2000 or HiSeq4000 instruments (Illumina, Inc., USA), resulting in >94% recovery at 10× coverage and >85% recovery at 20x coverage. GATK best practices pipeline was used for SNP and INDEL variant identification (http://www.broadinstitute.org/gatk/). Variants were annotated with in-house software1 and homozygous variant prioritization was done using custom Python scripts (available upon request) variant filtering.

Family 91

Exome sequencing was carried out with Nextera Rapid Capture Exome kit (Illumina) and sequenced on the HiSeq 2500 platform (Illumina). The resulting 100 bp paired-end sequence reads were mapped against the human reference genome assembly 19 (GRCh37) with the Burrows-Wheeler Aligner package 2 and read duplicates were removed with Picard (http://broadinstitute.github.io/picard/). Variant calling and indel realignments were performed with the Genome Analysis Toolkit (GATK) and variants were submitted to ANNOVAR for annotation 3.

Supplementary references

1. T. J. Dixon-Salazar, T.J et al. Exome sequencing can improve diagnosis and alter patient management. Sci. Transl. Med. 4, 138ra78 (2012).
2. Li, H et al. Fast and accurate short read alignment with Burrows-Wheeler transform. Bioinformatics (Oxford, England) *25* (14), 1754-60 (2019).
3. Wang, K et al. ANNOVAR: functional annotation of genetic variants from high-throughput sequencing data. Nucleic acids research**,** *38* (16), e164 (2010).
