# Supplementary material for: Loss of NARS1 impairs progenitor proliferation in cortical brain organoids and leads to microcephaly
Source: Nat Commun. 2020 Aug 12;11:4038. doi: 10.1038/s41467-020-17454-4 (PMC7424529; doi:10.1038/s41467-020-17454-4)
Supplement: Supplementary file 6 — Description of additonal sup files [file 41467_2020_17454_MOESM6_ESM.pdf]

Description of Supplementary Information

Title: Supplementary Data 1

Description: SNP genotyping assay and Karyotype results for all the iPSCs. Download separately.

Title: Supplementary Data 2

Description: Differential gene expression (DEG) of unaffected COs. Purple: indicates Homolog Gene ID and Term. Log transformed p-value, i.e. probability of seeing at least x number of genes out of the total number of genes in the list annotated to a particular GO term. Log transformed q value, i.e. statistical significance adjusted for the false discovery rate of 5%. Download separately.

Title: Supplementary Data 3

Description: Differential gene expression (DEG) of affected COs. Log transformed p-value i.e. probability of seeing at least x number of genes out of the total number of genes in the list annotated to a particular GO term. Log transformed q value, i.e. statistical significance adjusted for the false discovery rate of 5%. Download separately. Download separately.
