# Supplementary material for: Loss of NARS1 impairs progenitor proliferation in cortical brain organoids and leads to microcephaly
Source: Nat Commun. 2020 Aug 12;11:4038. doi: 10.1038/s41467-020-17454-4 (PMC7424529; doi:10.1038/s41467-020-17454-4)

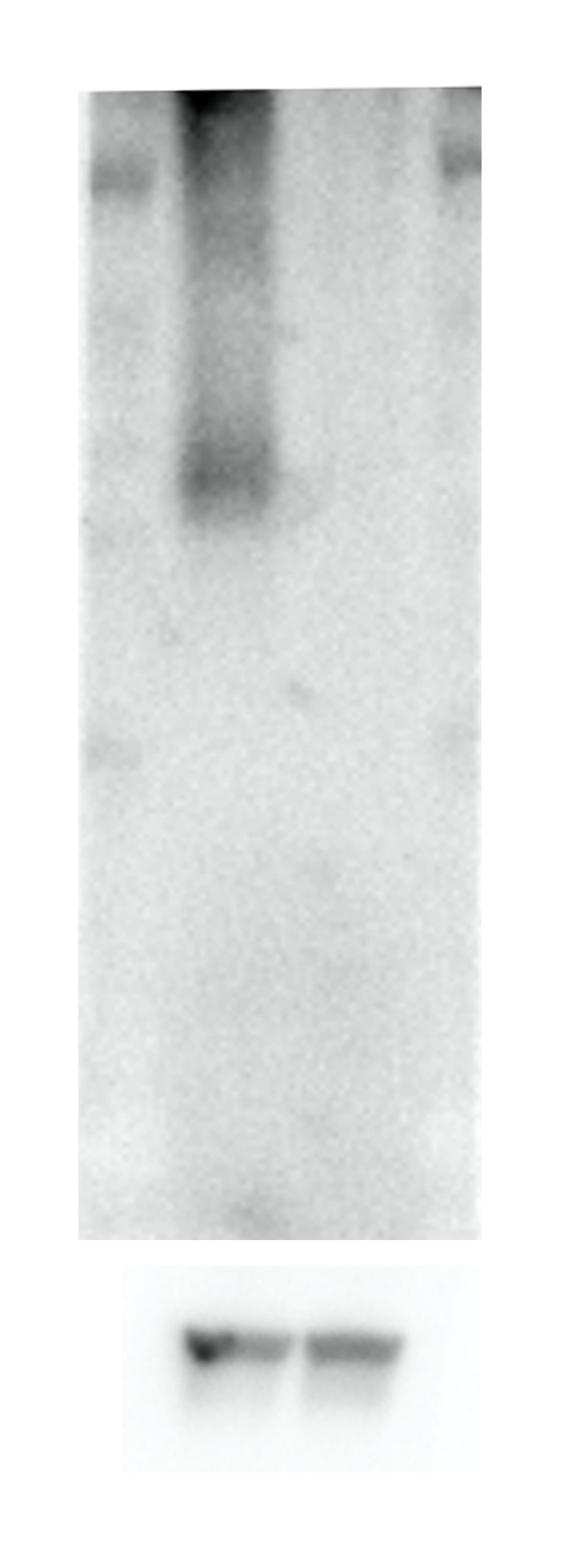
Uncropped Raw WB for NARS1 antibody

(See the legend in Supplementary Fig. 9)

Uncropped Raw WB for Fig2.a

(See the legend in Supplementary Fig.9)

Long-term exposure


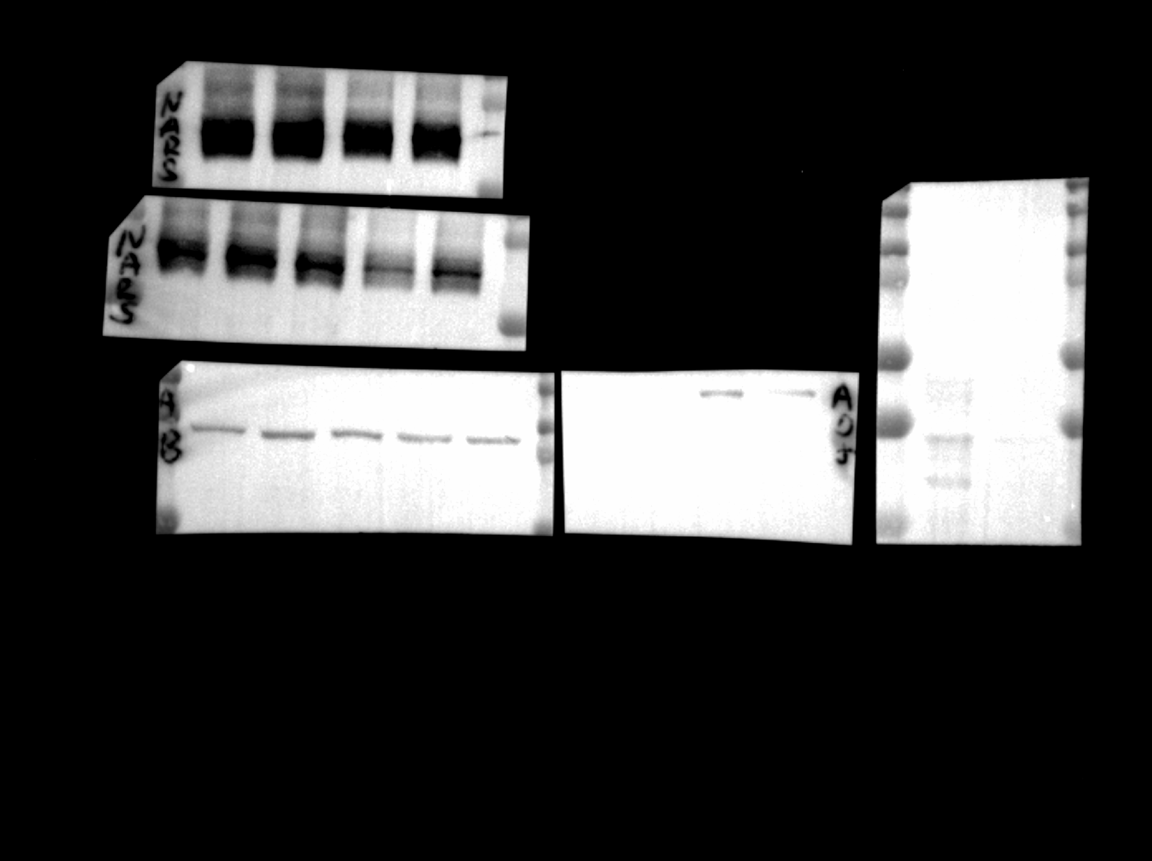


short-term exposure


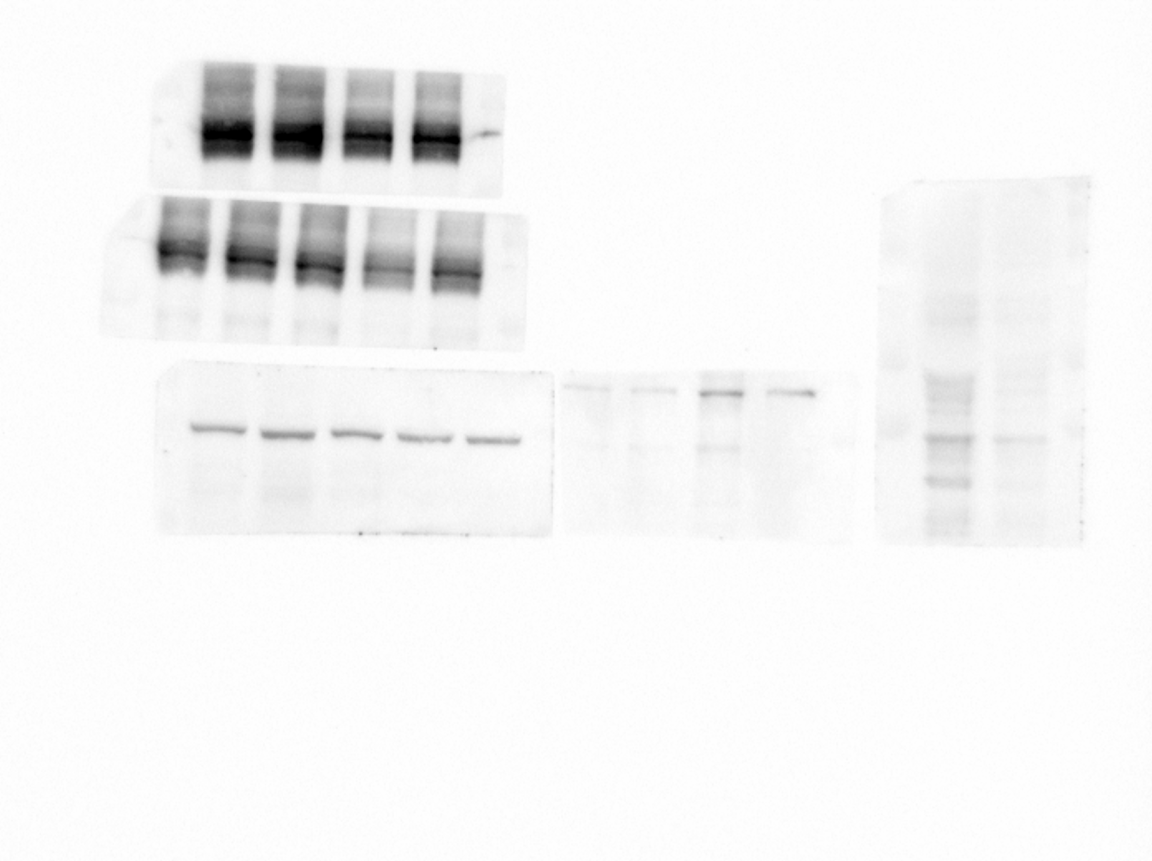


Uncropped Raw WB for Fig2.c

(See the legend in Supplementary Fig.9)

Long-term exposure short-term exposure


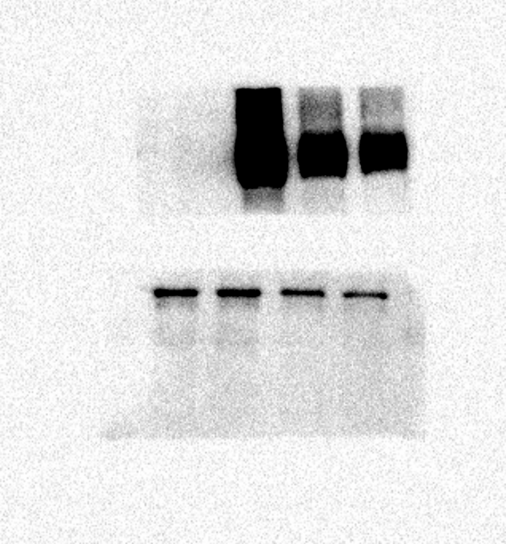


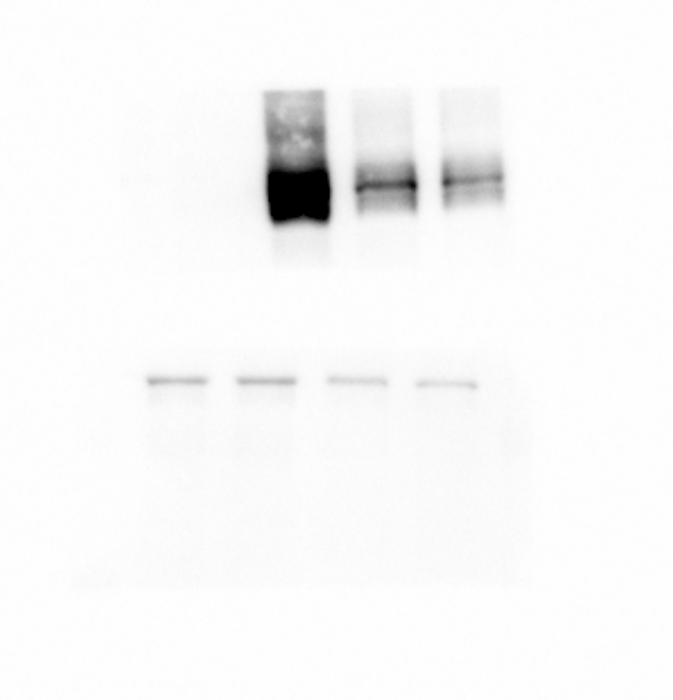


Uncropped Raw WB for Fig2.f

(See the legend in Supplementary Fig.9)

Replicate 1 Replicate 2


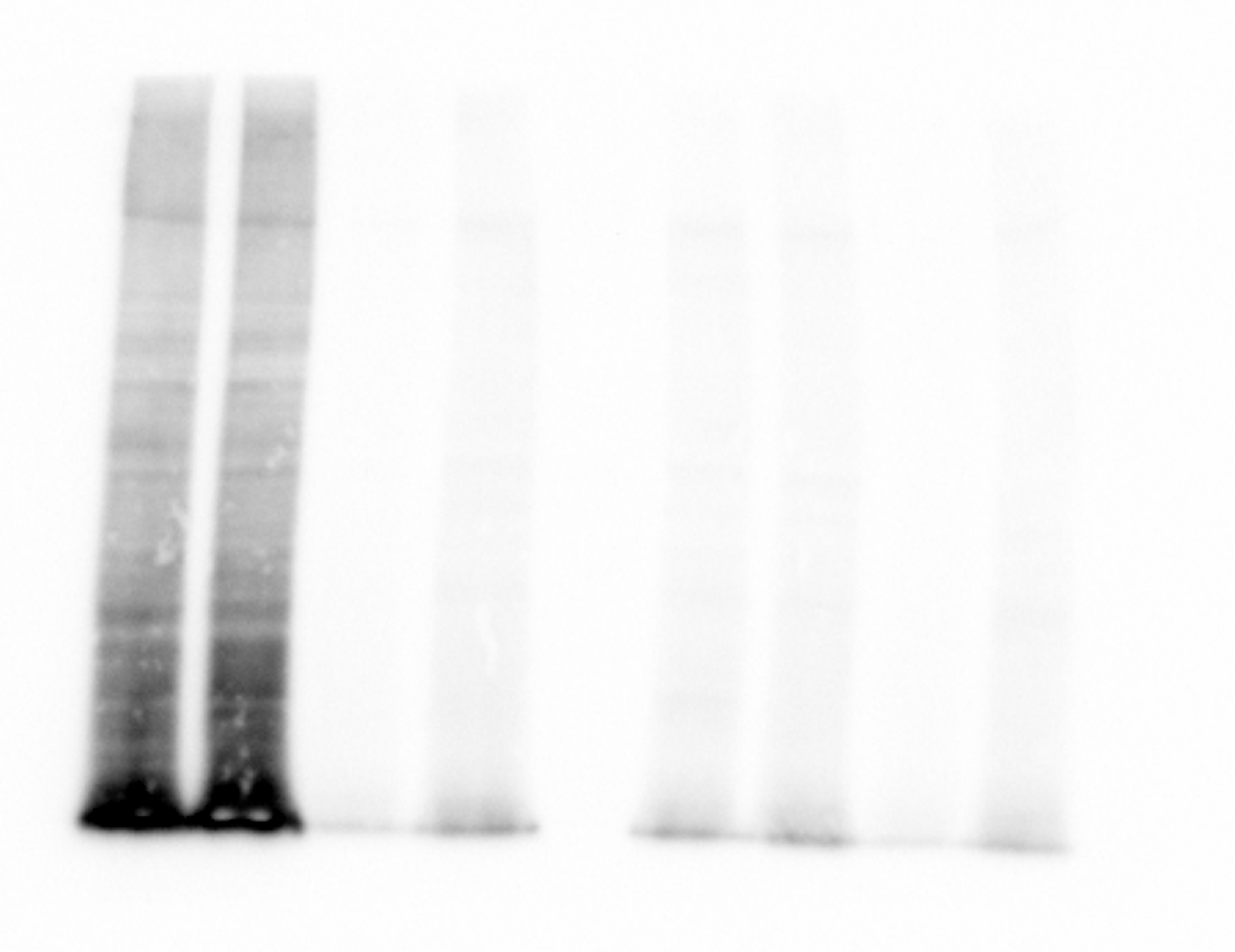


Uncropped Raw WB for Fig5.c

(See the legend in Supplementary Fig.9)


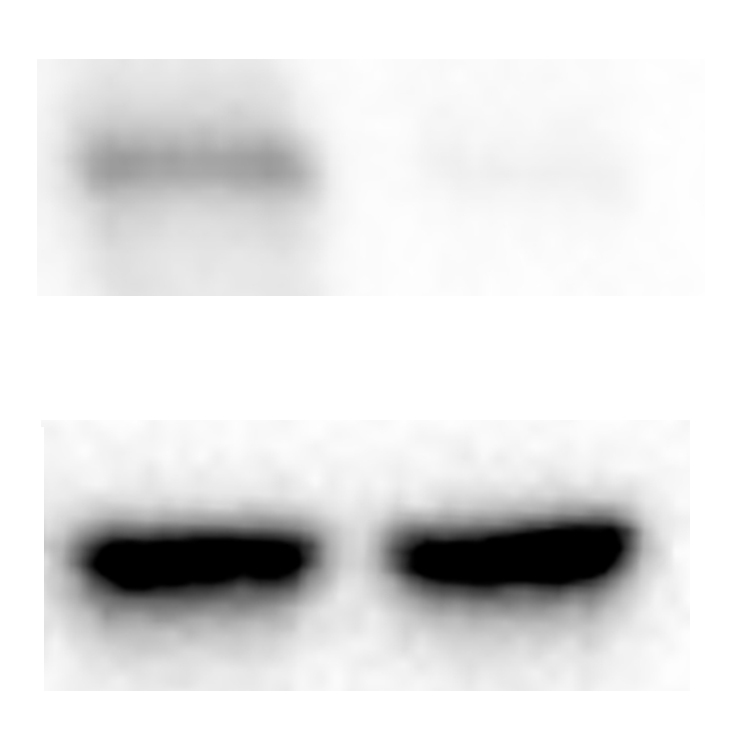


Uncropped Raw WB for Supplementary Fig 2. a

(See the legend in Supplementary Fig.9)


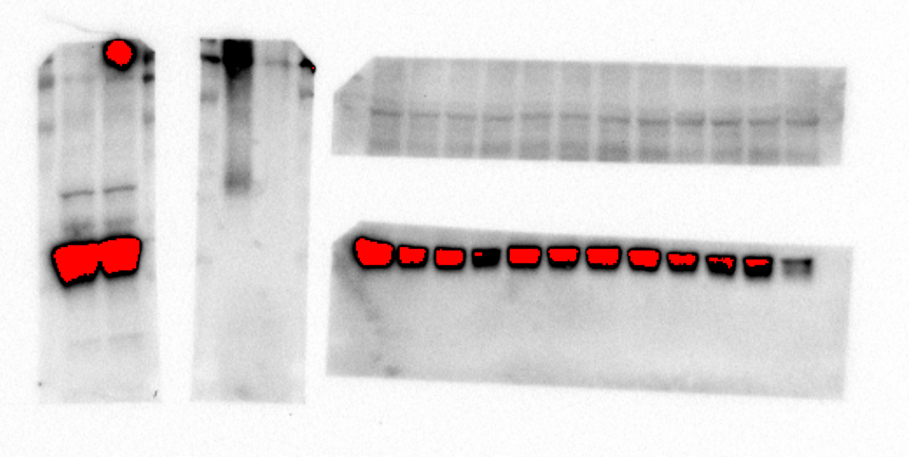


Long-term exposure


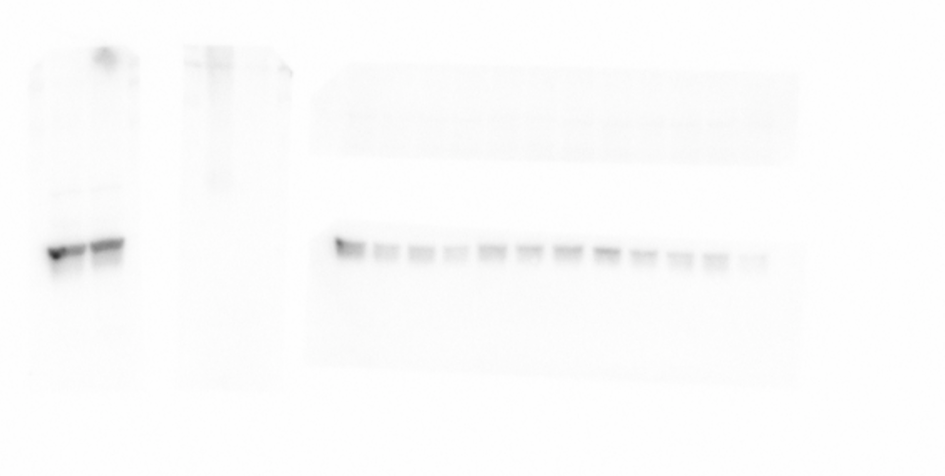


short-term exposure

Uncropped Raw WB for Supplementary Fig 2. c


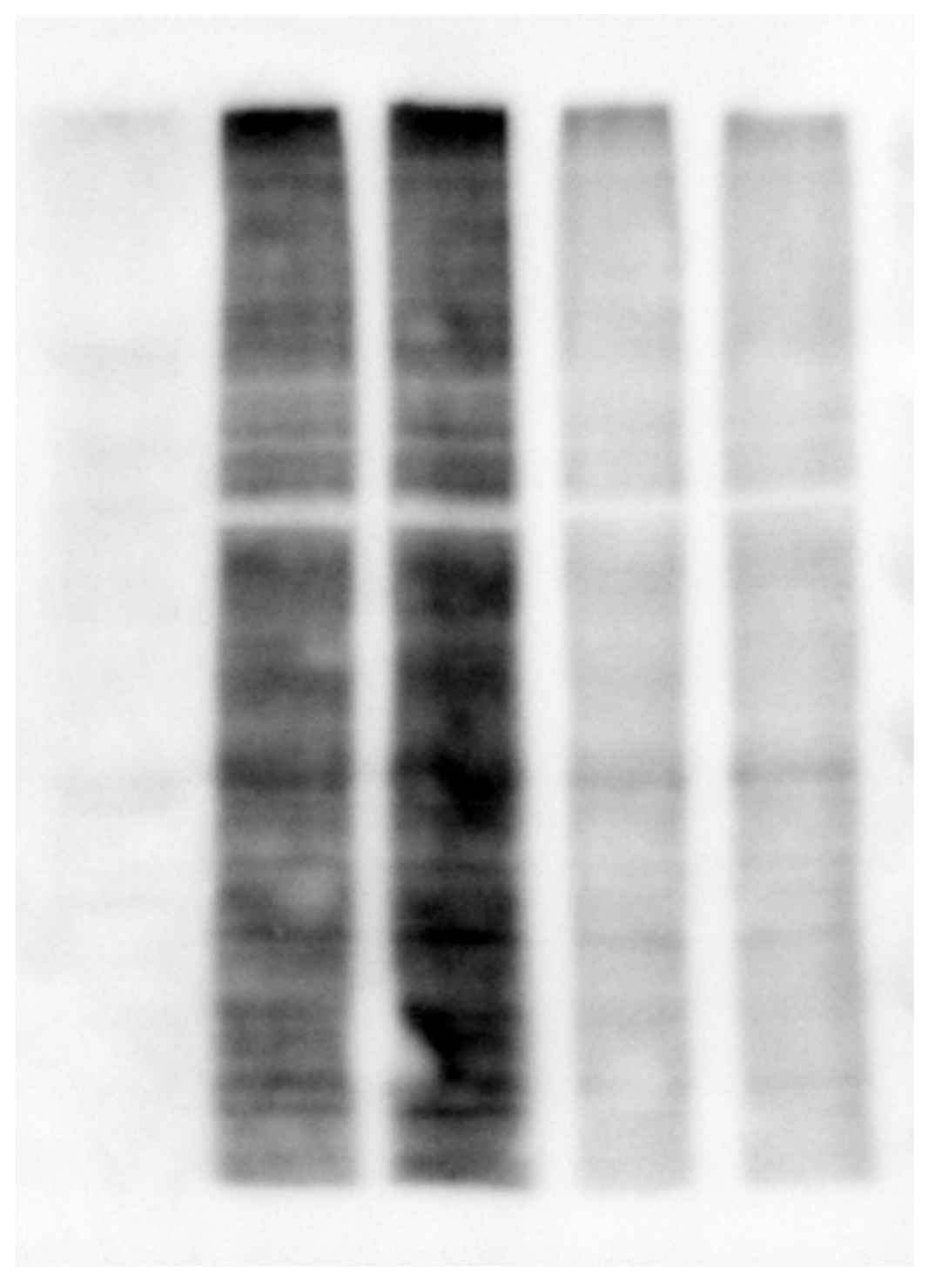
(See the legend in Supplementary Fig.9)


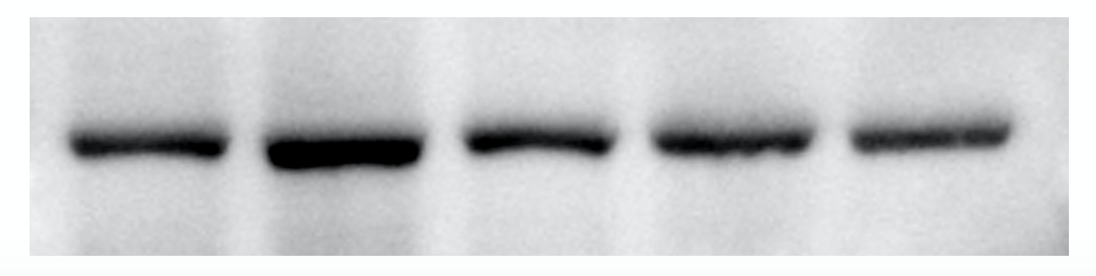

Supplement: Supplementary file 8 — Source Data 1 [file 41467_2020_17454_MOESM8_ESM.docx]
